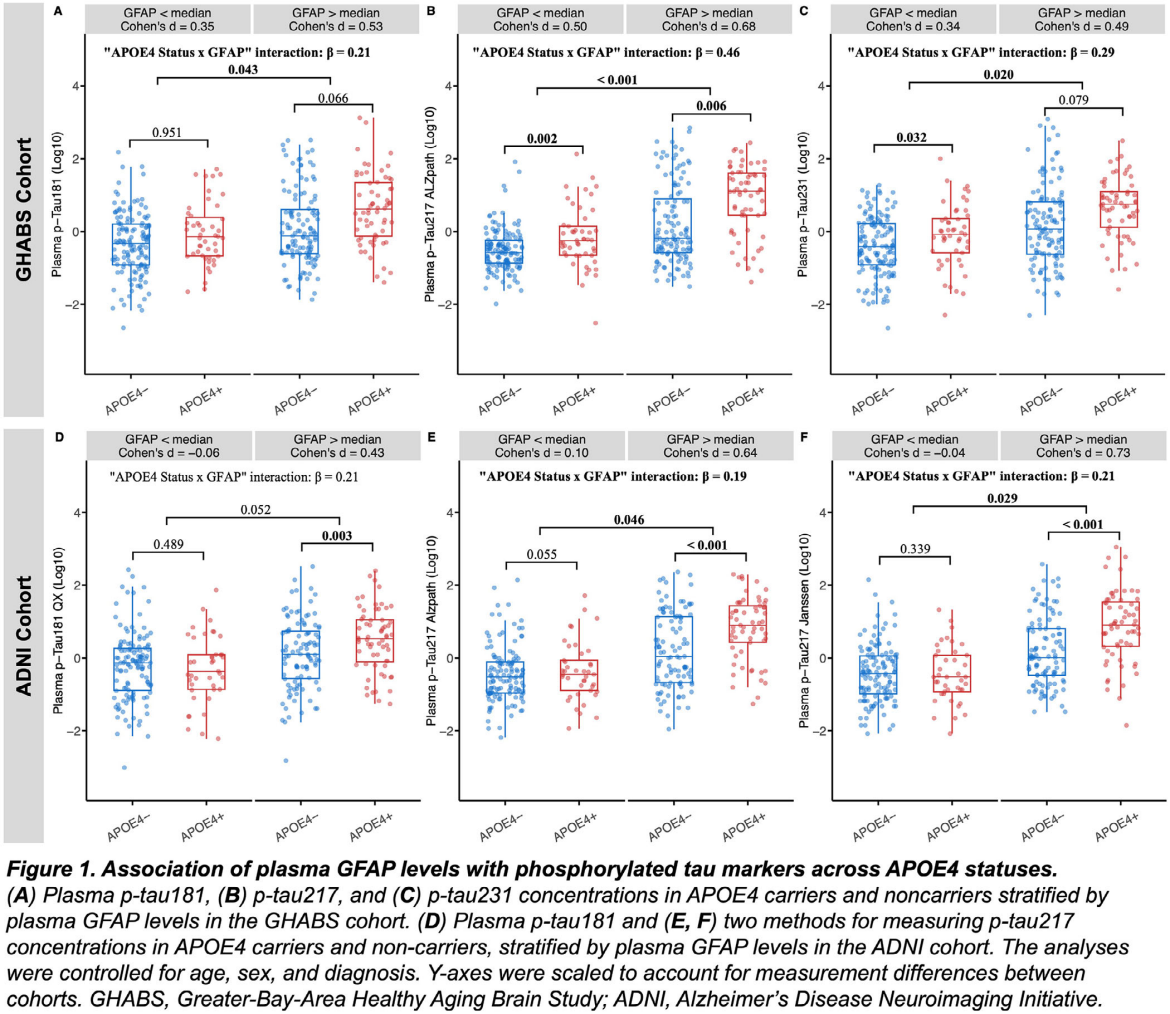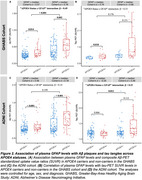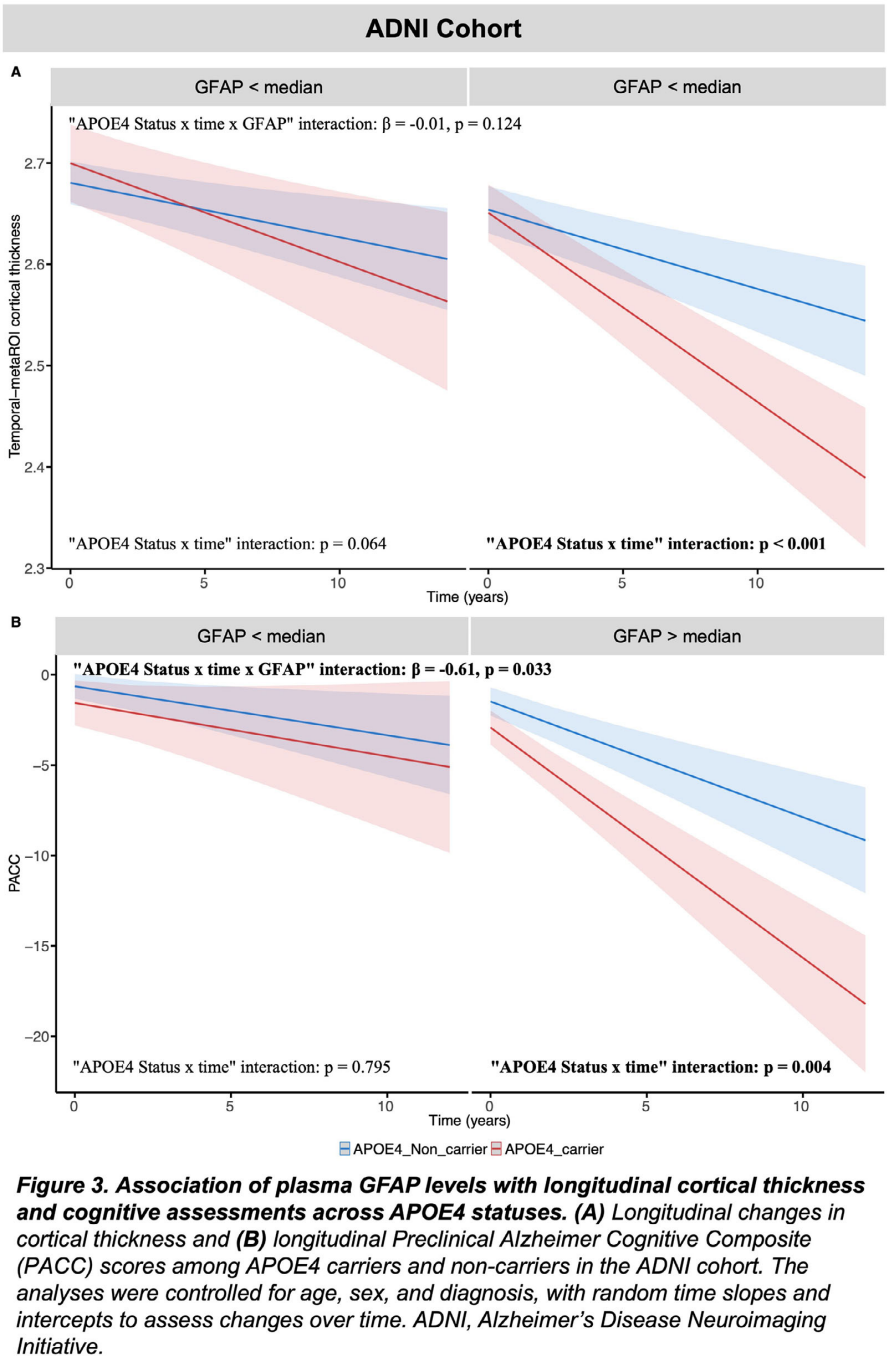# Plasma glial fibrillary acidic protein correlates with APOE4‐associated Alzheimer's pathological changes

**DOI:** 10.1002/alz70856_106216

**Published:** 2026-01-07

**Authors:** Yalin Zhu, Laihong Zhang, Mingxing Jiang, Anqi Li, Jie Yang, Lili Fang, Jieyin li, Guoyu Lan, Guojun Bu, Tengfei Guo

**Affiliations:** ^1^ The Hong Kong University of Science and Technology, Hongkong, Hongkong, China; ^2^ Shenzhen Bay Laboratory, Shenzhen, Guangdong, China; ^3^ Institute of Neurological and Psychiatric Disorders, Shenzhen Bay Laboratory, Shenzhen, Guangdong, China; ^4^ Xuanwu Hospital of Capital Medical University, Beijing, Beijing, China; ^5^ Tsinghua Shenzhen International Graduate School (SIGS), Tsinghua University, Shenzhen, China; ^6^ Peking University Shenzhen Graduate School, Shenzhen, Guangdong, China

## Abstract

**Background:**

Apolipoprotein E (APOE) is a well‐established genetic risk factor for Alzheimer's disease (AD). Plasma glial fibrillary acidic protein (GFAP) is a key biomarker of neuroinflammation. However, the association of plasma GFAP with the relationship between APOE and AD pathology has not yet been thoroughly investigated. Therefore, this study aims to explore the association of plasma GFAP levels with APOE genotypes, amyloid‐β (Aβ) accumulation, tau pathology, cortical thickness, and cognitive decline.

**Method:**

The study involved 706 participants from the GHABS and ADNI cohorts, with measurements of plasma GFAP, phosphorylated tau markers (*p*‐tau181 and *p*‐tau217 for both cohorts, and *p*‐tau231 available only for GHABS), Aβ‐PET and tau‐PET imaging. A subset of 336 individuals from the ADNI had follow‐up structural MRI scans and cognitive assessments. We investigated the interaction of plasma GFAP and APOE genotypes in Aβ plaques and tau tangles using generalized linear models (GLM). Longitudinal analyses of cortical thickness and cognitive decline in the ADNI cohort were conducted using linear mixed‐effects (LME) models, stratifying participants into “GFAP > Median” and “GFAP < Median” groups based on their plasma GFAP level. Models controlled for age, sex, and diagnosis, with random time slopes and intercepts to assess changes over time.

**Result:**

Individuals with higher plasma GFAP levels (> Median) exhibited higher plasma concentrations of *p*‐tau181 and *p*‐tau217 in APOE4 carriers across both cohorts compared to those with lower GFAP levels (< Median), as well as a significantly higher level in *p*‐tau231 was observed in APOE4 carriers within the GHABS cohort (Figure 1). Additionally, elevated GFAP levels were linked to increased Aβ and tau accumulation in APOE4 carriers (Figure 2). Furthermore, higher plasma GFAP levels correlated with decreased longitudinal cortical thickness and PACC scores in APOE4 carriers within the ADNI cohort (Figure 3).

**Conclusion:**

This study highlights the association between plasma GFAP levels and AD pathology in relation to APOE genotypes. Elevated plasma GFAP levels are associated with more severe AD‐related pathologies, particularly in APOE4 carriers. These findings emphasize the potential of GFAP as a biomarker for astrocytic activation and its relationship with genetic susceptibility in AD progression.